# Whole genome amplification of degraded and nondegraded DNA for forensic purposes

**DOI:** 10.1007/s00414-012-0764-9

**Published:** 2012-09-01

**Authors:** Agnieszka Maciejewska, Joanna Jakubowska, Ryszard Pawłowski

**Affiliations:** 1 Institute of Forensic Medicine, Medical University of Gdańsk, Dębowa 23, 80-204 Gdańsk, Poland; 2 Intercollegiate Faculty of Biotechnology, University of Gdańsk and Medical University of Gdańsk, Kładki 24, 80-822 Gdańsk, Poland

**Keywords:** Whole genome amplification, Degraded DNA, STR typing, mtDNA typing, Y-STR typing, Forensic genetics

## Abstract

Degraded DNA is often analyzed in forensic genetics laboratories. Reliable analysis of degraded DNA is of great importance, since its results impact the quality and reliability of expert testimonies. Recently, a number of whole genome amplification (WGA) methods have been proposed as preamplification tools. They work on the premise of being able to generate microgram quantities of DNA from as little as the quantity of DNA from a single cell. We chose, investigated, and compared seven WGA methods to evaluate their ability to “recover” degraded and nondegraded DNA: degenerate oligonucleotide-primed PCR, primer extension preamplification PCR, GenomePlex™ WGA commercial kit (Sigma), multiple displacement amplification, GenomiPhi™ Amplification kit (Amersham Biosciences), restriction and circularization-aided rolling circle amplification, and blunt-end ligation-mediated WGA. The efficiency and reliability of those methods were analyzed and compared using SGMPlus, YFiler, mtDNA, and Y-chromosome SNP typing. The best results for nondegraded DNA were obtained with GenomiPhi and PEP methods. In the case of degraded DNA (200 bp), the best results were obtained with GenomePlex which successfully amplified also severely degraded DNA (100 bp), thus enabling correct typing of mtDNA and Y-SNP loci. WGA may be very useful in analysis of low copy number DNA or degraded DNA in forensic genetics, especially after introduction of some improvements (sample pooling and replicate DNA typing).

**Electronic supplementary material:**

The online version of this article (doi:10.1007/s00414-012-0764-9) contains supplementary material, which is available to authorized users.

## Introduction

One of the major problems of forensic genetics is the difficulty in analysis of highly degraded DNA or low copy number (LCN) DNA. LCN DNA is arbitrary defined as less than 100 pg of template [1]. Small number of template copies and the availability of only short DNA fragments result in lack of amplification of some DNA fragments. Consequently, locus and allele dropouts, which are stochastic effect symptoms [2], disturb proper analysis of biological traces.

Two main approaches are used to solve the problem of degraded or LCN DNA samples: analysis of mtDNA [3–5] or increasing the number of PCR cycles [1]. These actions often do not produce the desired effect, mainly because of very low discriminatory power of mtDNA and artifacts (additional fractions, contamination, loss of alleles, and loci) which can falsify DNA profiles when increased number of cycles is used [6].

Another solution is to use whole genome amplification (WGA) method, which preamplifies DNA before final PCR typing. Recently, several WGA techniques and their modifications have been described: primer extension preamplification (PEP) [7–10], degenerate oligonucleotide-primed PCR (DOP-PCR) [11], rolling circle amplification (RCA) [12], multiple displacement amplification (MDA) [13], restriction and circularization-aided rolling circle amplification (RCA-RCA) [14], and blunt-end ligation-mediated WGA (BL-WGA) [15]. Despite technical differences, all these methods work on the premise of being able to generate microgram quantities of DNA from as little as the quantity of DNA present in a single cell. WGA methods are especially applicable in medical diagnosis which includes cancer analysis, preimplantation genetic diagnosis, prenatal diagnosis, and study of human malignancies [16]. Only few of them have been analyzed as a tool in forensics. Moreover, the existing analyses focused rather on LCN DNA [10, 17–20] than degraded DNA [18, 20, 21], which usually requires different approaches.

We chose, investigated, and compared seven WGA methods to evaluate their ability to amplify degraded and nondegraded DNA samples. They included PCR-based techniques: DOP-PCR [11], PEP-PCR [8], GenomePlex™ WGA commercial kit (Sigma), as well as isothermal reaction-based methods which utilize highly precise Phi29 polymerase with strand displacement activity: MDA [13], its commercial version GenomiPhi™ Amplification kit (Amersham Biosciences), RCA-RCA [14], and BL-WGA—the MDA modification utilizing T4 ligase and T4 polymerase [15]. To our best knowledge, this is the first paper comparing and analyzing different WGA methods used to reactivate heavily degraded DNA in forensic genetics.

## Materials and methods

### Material

We used human muscle tissue samples collected from male thigh during autopsy (24 h after death), and 4-year-old, formalin-fixed, paraffin-embedded (FFPE) muscle tissue samples containing naturally degraded DNA with high degree of degradation (ca, 100 bp). For tissue fixation, unbuffered formalin was used.

### Biological DNA degradation and estimation of the degradation rate

Tissue sample (ca, 10 g) was incubated in a sealed tube at 56 °C in a shaking water bath until the DNA reached the size of 200 bp (38 days of degradation) or 100 bp (47 days of degradation). The degraded tissue samples were then subjected to proteinase K/phenol–chloroform DNA isolation. The DNA degradation process was controlled, initially every week, and later every 2–3 days of tissue incubation, using 0.5 % agarose gel electrophoresis with subsequent staining with SYBR Green I and pGEM (Promega) as size marker. Additionally, real-time PCR, using Quantifiler Human kit (Applied Biosystems, USA) and ABI PRISM 7900 HT Real-Time Fast PCR System, was used. Moreover, the degradation degree was confirmed by amplification of the loci present in SGMPlus™ and YFiler™ kits (Applied Biosystems, USA). The range of DNA amplicons in the analyzed samples was from 67 to 469 bp.

### DNA isolation

Fifteen milligrams of muscle tissue was incubated with 636 μl TE9 buffer (0.5 M Tris, 0.02 M EDTA, 0.01 M NaCl, and pH 9.0), 314 μl proteinase K (2 mg/ml), and 50 μl 20 % SDS overnight at 56 °C. DNA was extracted with phenol–chloroform–isoamyl alcohol (25:24:1) and precipitated with ethanol and 3 M sodium acetate. The DNA from nondegraded muscle samples showed high level of polymerization (supplementary material, Figs. S1 and S2).

Five-micrometer slice of formalin-fixed, paraffin-embedded tissue (FFPE) was placed in a 1.5-ml tube with 500 μl of xylene, vortexed, and spinned down (12,000 rpm/5 min). The pellet was washed twice with 200 μl of absolute ethanol. Digestion, extraction, and precipitation were carried out as described above for muscle tissue.

### Whole genome amplification

The DNA originating from degraded and nondegraded muscle tissues was subjected to amplification using seven WGA methods: DOP-PCR [11], PEP-PCR [8], GenomePlex™ WGA (Sigma), MDA [13], GenomiPhi DNA Amplification kit (Amersham Biosciences), RCA-RCA [14], and BL-WGA [15], as recommended by researchers (BL-WGA) or manufacturers (GenomiPhi and GenomePlex) or with some modifications (Table 1). The methods were applied after a detailed validation which aimed at improving their utility (data not shown). In most experiments, 100, 10, or 1 ng of degraded and nondegraded DNA was used. In some cases, 0.5, 0.25, and 0.125 ng of nondegraded DNA was subjected to WGA preamplification.

**Table 1:** Modifications of PEP, DOP, MDA, and RCA-RCA protocols

PEP			DOP			MDA			RCA-RCA		
Parameter	Zhang et al. [8] protocol	Modified PEP protocol	Parameter	Telenius et al. [11] protocol	Modified DOP protocol	Parameter	Dean et al. [13] protocol	Modified MDA protocol	Parameter	Wang et al. [14] protocol	Modified RCA-RCA protocol
Polymerase	Taq Polymerase	AmpliTaq Gold Polymerase (Applied Biosystems)	Polymerase	Taq Polymerase	JumpStart Taq Polymerase (Sigma)	Φ 29 polymerase amount/reaction volume	80 U/100 μl	6 U/10 μl	Circularization, T4 DNA ligase amount/reaction volume	1,000 U/15 μl	1,350 U/15 μl
Polymerase amount/reaction volume	5 U/60 μl	2.5 U/20 μl	Polymerase amount/reaction volume	1.25U/50 μl	5U/50 μl	Yeast pyrophosphatase	1 U/ml	Not used	Amplification, Φ 29 polymerase amount/reaction volume	3 U/20 μl	8 U/10 μl
Temperature profile	50 cycles (92 °C/1 min, 37 °C/2 min, ramping step 1 °C/10 s to 55 °C, 55 °C/4 min)	95 °C/11 min	Temperature profile	95 °C/5 min	95 °C/3 min						
		45 cycles (92 °C/1 min, 25 °C/2 min, ramping step 1 °C/1 s to 60 °C, 60 °C /4 min)		5 cycles (94 °C/1 min, 30 °C/1 and 5 min, 3-min transition 30–72 °C, 72 °C/3 min)	5 cycles (94 °C/1 min, 25 °C/1 and 5 min, ramping step 0.2 °C/1 s to 72 °C, 72 °C/3 min)						
		68 °C/10 min		35 cycles (94 °C/1 min, 62 °C/1 min, 72 °C/3 min)	40 cycles (94 °C/1 min, 64 °C/1 min, 72 °C/3 min)						
				72 °C/10 min	72 °C/10 min						

### Quantification of DNA

Quantification of DNA before and after WGA reaction was carried out using Fluoroskan Ascent FL (ThermoScientific) with PicoGreen® or using 7900HT Real-Time Fast PCR System (Applied Biosystems).

### Polymorphic loci amplification and evaluation of electrophorograms

To amplify short tandem repeats (STR), two commercial multiplex PCR kits were used: SGMPlus™ (Applied Biosystems) for autosomal polymorphic loci and YFiler™ (Applied Biosystems) for Y-chromosome polymorphic loci, as recommended by the manufacturer. HVI and HVII regions of mtDNA were sequenced with BigDye® Terminator v1.1 Cycle Sequencing kit (Applied Biosystems), as recommended by the manufacturer. The following primers were used to amplify HVI and HVII regions: F15971 and R16410 and F15 and R484, respectively.

Y-chromosome SNP typing of M173, M9, M35, and YAP loci was performed, as described by Kayser et al. [22], using PCR-RFLP or SNaPshot method (Applied Biosystems). The products were separated using polyacrylamide electrophoresis and silver staining, as described in Pawłowski et al. [23].

The detection of products was carried out using an ABI PRISM® 3130 or 310 Genetic Analyzer (Applied Biosystems) and analyzed with GeneMapper v. 3.1 and GeneScan v. 3.7 software, respectively. Sequence analysis was carried out using an ABI PRISM® 3130, as well as Sequencing Analysis v. 5.2 and SeqScape v. 2.5 computer programs.

## Results and discussion

### Degradation of fresh muscle tissue

Analysis of degraded DNA is essential in terms of WGA use. It is very common that degraded, low-quality, and low copy number DNA is analyzed in forensic genetics laboratories. The currently used DNA typing methods, although very sensitive, are, in many cases, not good enough to successfully type difficult samples. For this reason, reliable methods enabling analysis of this kind of DNA are extremely important. DNA of 100–200 bp mimics DNA degraded by environmental factors. In our experiments, muscle tissue was subjected to biological in vitro degradation until the DNA was degraded to ca 200 or 100 bp (supplementary material, Figs. S1 and S2).

### Comparison of the yield of amplification of WGA methods for nondegraded and degraded DNA

The DNA isolated from native and degraded (200 bp) muscle tissues was amplified using seven different WGA methods. Preamplification analysis was performed using 1, 10, and 100 ng of input.

The highest increase in DNA amount was observed for RCA-RCA method for both degraded and nondegraded DNA, reaching even 12,000-fold increase for 1 ng input of nondegraded DNA (Table 2). Similar increase was observed for BL-WGA method. The lowest amplification efficiency was observed for PEP method.

**Table 2:** Increase in DNA amount observed for 100, 10, and 1 ng of nondegraded and degraded DNA preamplified with different WGA techniques

DNA input (ng)		PEP	DOP	MDA	GenomiPhi™	GenomePlex™	RCA-RCA	BL-WGA
100	ND	12×	28×	73×	40×	290×	941×	244×
	D	11×	15×	29×	38×	120×	852×	197×
10	ND	78×	410×	1,022×	810×	918×	7,970×	2,636×
	D	25×	236×	423×	334×	324×	4,995×	1,978×
1	ND	395×	5,620×	10,034×	3,619×	153×	12,043×	9,049×
	D	144×	2,032×	796×	2,286×	75×	10,051×	8,752×

*ND* nondegraded DNA, *D* degraded DNA

As shown in Table 2, the increase in DNA amount after WGA amplification was usually inversely proportional to the initial DNA concentration. The exception was GenomePlex technique which produced the highest increase for 10 ng of DNA. However, this was consistent with manufacturer’s information (http://wwwsigmaaldrichcom/wga). In all cases, the increase observed in degraded DNA samples was similar but lower than the increase in the samples containing equal amounts of nondegraded DNA. When 1 ng input of DNA was used, the most significant difference between the increase of degraded and nondegraded DNA amounts was observed for MDA technique, while the least significant difference was observed for BL-WGA and RCA-RCA. When higher DNA concentrations were used, the observed differences between degraded and nondegraded DNA samples were less significant. Probably, the main cause is the excess of substrate added to the reaction mixture. RCA-RCA and BL-WGA also showed the highest increase in degraded DNA amount, while PEP and GenomePlex produced the lowest yield (Table 2). It could be explained by different mechanisms of the methods. MDA operates on long DNA templates, and its efficiency diminishes with the decrease of size of DNA strands [24]. In contrast, the principle of RCA-RCA is fragmentation of genome using restriction enzymes and circularization preceding the MDA-based amplification, which is beneficial for amplification of short fragments [14].

Our results did not show a significant difference between the efficiency of RCA-RCA and BL-WGA for DNA degraded to 200 bp. However, it was suggested that RCA-RCA may not allow efficient amplification of fragments shorter than 250 bp [14, 25], while BL-RCA allows amplification of even small fragments of DNA (ca, 200 bp), thanks to additional steps: conversion of DNA fragments to blunt-ends by T4 DNA polymerase and self-ligation or cross-ligation by T4 DNA ligase [15].

The results obtained for nondegraded DNA were usually consistent with the results obtained by other investigators who used GenomiPhi, PEP, DOP, MDA, RCA-RCA, and BL-WGA [13-15, 18, 26]. In some cases, they may slightly differ, i.e., other investigators obtained about ten times more DNA with GenomePlex than we did [18].

Only a few studies have been performed to analyze the influence of WGA on the increase of degraded DNA amount, and the researchers focused rather on the possibility of obtaining STR profiles or recovery of other features [10, 14, 18, 20]. The increase of DNA amount is one of the main characteristics of WGA methods. However, a high amount of DNA does not ensure good quality of a DNA profile. In our practice, we encounter tissues that, despite a high concentration of DNA, show very strong degree of its degradation. In the case of nondegraded DNA, low amounts of DNA are sufficient to get reliable results, but for severely degraded DNA samples, usually high amounts of DNA are needed. Thus, we also decided to preamplify the DNA up to very high concentrations (100 ng).

### Analysis of STR profiles of DNA preamplified using WGA

To evaluate the quality of WGA-preamplified DNA, samples of native DNA, degraded DNA (200 bp), and WGA-preamplified DNA were tested using SGMPlus kit. The obtained profiles were subjected to detailed evaluation, especially in terms of: accordance with native profiles, locus and allele dropouts, allelic imbalance, artifacts, and differences between the most and the least efficiently amplified loci.

Table 3 presents the impact of the amount of preamplified DNA on quality of SGMPlus profiles when using different WGA methods. Significant differences were observed between the cases of successful amplification of degraded and nondegraded WGA-preamplified DNA samples.

**Table 3:** Impact of the amount of preamplified DNA on the quality of SGMPlus profiles when using different WGA methods

DNA input (ng)	Sample	PEP				DOP				MDA				GenomiPhi™				GenomePlex™				RCA-RCA				BL-WGA			
		AR (%)	Add peaks	HI (%)	ES (%)	AR (%)	Add peaks	HI (%)	ES (%)	AR (%)	Add peaks	HI (%)	ES (%)	AR (%)	Add peaks	HI (%)	ES (%)	AR (%)	Add peaks	HI (%)	ES (%)	AR (%)	Add peaks	HI (%)	ES (%)	AR (%)	Add peaks	HI (%)	ES (%)
100	ND	100	−	>60	−	100	−	29	−	95.2	−	49	−	100	−	54	−	80.9	−	55	26	95.2	+	10	−	85.7	+	26	−
	D	90.4	−	29	−	0	−	−	−	0	−	−	−	0	−	−	−	90.4	+	24	22	76.2	+	41	−	42.8	+	26	−
10	ND	100	−	>60	−	100	−	49	−	90.4	−	48	−	100	−	41	−	71.4	+	56	37	76.6	−	19	−	38.0	−	56	−
	D	14.2	−	−	−	0	−	−	−	0	−	−	−	0	−	−	−	23.8	−	29	−	9.5	−	−	−	52.3	−	9	−
1	ND	100	−	>60	−	100	−	50	−	95.2	−	49	−	100	−	53	−	80.9	+	55	48	14.2	−	47	−	4.7	−	−	−
	D	4.7	−	−	−	0	−	−	−	0	−	−	−	0	−	−	−	19	−	33	−	0	−	−	−	0	−	−	−
0.5	ND	95.2	NA	NA	NA	NA	NA	NA	NA	NA	NA	NA	NA	NA	NA	NA	NA	NA	NA	NA	NA	NA	NA	NA	NA	NA	NA	NA	NA
0.25	ND	71.4	NA	NA	NA	NA	NA	NA	NA	NA	NA	NA	NA	NA	NA	NA	NA	NA	NA	NA	NA	NA	NA	NA	NA	NA	NA	NA	NA
0.125	ND	42.5	NA	NA	NA	NA	NA	NA	NA	NA	NA	NA	NA	NA	NA	NA	NA	NA	NA	NA	NA	NA	NA	NA	NA	NA	NA	NA	NA

Alleles recovery calculated as a ratio of the number of amplified loci after WGA preamplification, to the number of alleles present in a nondegraded sample (in percent). In the nondegraded sample, 21 alleles were present (100 %). Alleles with RFU of >50 were counted. Additional peaks (artifacts), not consistent with the lengths of the known alleles present in allelic ladders; present (+), absent (−). HI > 60 % was considered as an acceptable ratio

*AR* alleles recovery, *Add peaks* additional peaks, *HI* the highest heterozygote’s imbalance observed (in percent), *ES* the highest elevated stutter expressed as a ratio of n-4 area to n allele area (in percent), *ND* nondegraded DNA, *D* degraded DNA, *NA* not analyzed

Complete amplification of all loci of nondegraded DNA without allele dropouts and drop-ins occurred for PEP, DOP, and GenomiPhi, while for MDA, GenomePlex, RCA-RCA, and BL-RCA, both locus and allele dropouts were observed. In the case of those methods, some loci, unlike others present in SGMPlus, are extremely preferentially amplified (by orders of magnitude), which results in dropouts. Extra alleles, which were absent in native profile, appeared in GenomePlex profiles (1 and 10 ng of DNA), as well as in RCA-RCA, and BL-WGA profiles (100 ng of DNA). Moreover, for GenomePlex (1 ng) and BL-RCA (100 ng), amplicons were amplified, which lengths were inconsistent to the lengths of known alleles (artifacts). The only method, which produced remarkably elevated amount of stutters, was GenomePlex for which n-4 and n-8 stutters were observed. The highest ratio of elevated n-4 stutters to the n fraction was 48 % and was decreasing with the increase of template DNA amount.

Most of the WGA methods produced imbalanced heterozygotes with the lowest ratios of 40–50 %. An extreme heterozygote imbalance was observed for RCA-RCA preamplification. The strongest imbalance was observed for 100 ng input DNA, strong for 10 ng and low but the most acceptable for 1 ng of input DNA (Table 3). For 100 ng of DNA, strong heterozygote imbalance was also observed for DOP and BL-WGA methods. It was observed that the increase of input DNA caused the higher heterozygote imbalance. Although it seems unusual, this was probably caused by the fact that, in the case of RCA-based methods, the increase of DNA amount enabled amplification of loci which, in lower concentrations, were not successfully amplified (locus or allele dropouts were observed). There are some serious implications of imbalanced alleles. It can lead, for instance, to wrong interpretation of profiles originating from a single source, such as a DNA mixture. The only method which produced correct heterozygote ratios for nondegraded DNA was PEP (Table 3).

Among the methods for which no locus and allele dropouts or additional fractions occurred (PEP, DOP, and GenomiPhi), the most balanced profiles were obtained in the case of GenomiPhi. The maximum difference between the least and the most amplified locus was five- to sevenfold. Generally, for almost all WGA methods, the increase in template amount led to the increase of allelic amplification efficiency ratio, thereby giving the largest difference for 100 ng of template DNA. The analysis of DNA profiles obtained with different WGA methods revealed that the most efficiently amplified loci were: D8S1179 in three cases (DOP, MDA, and GenomePlex), VWA in two cases (PEP and GenomiPhi), and D16S539 in the remaining two cases (RCA-RCA and BL-RCA).

PEP produced pretty good results for nondegraded DNA, so we chose this method to analyze the effect of further DNA amount reduction on SGMPlus profile completeness. The range of 100 to 0.125 ng of template DNA was analyzed. For amounts less than 1 ng, we observed allele dropouts. For amounts of 0.5, 0.25, and 0.125 ng, we obtained 95.2, 71.4, and 42.8 % of full profile allelic coverage respectively.

**Fig. 1:**
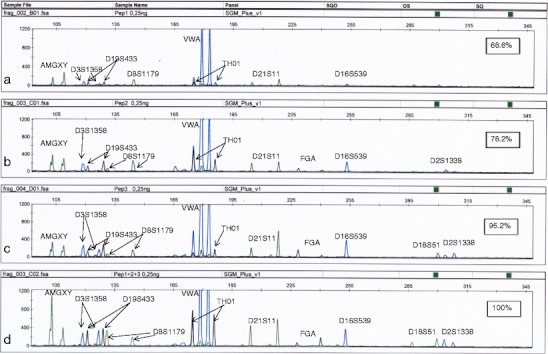
SGMPlus profiles of triple PEP preamplifications of the same DNA extract and pooled products. PEP was run with 0.25 ng of template. *Numbers* indicate the percentage of successfully amplified alleles. **a**, **b**, **c** SGMPlus profiles of single, independent PEP reactions. **d** SGMPlus profile of pooled products

Since we obtained slightly different profiles with different PEP preamplifications, we decided to investigate whether pooling of single PEP reactions would increase alleles’ recovery. Three separate PEP reactions and SGM amplifications were run and pooled. Pooling of products of three reactions was very beneficial for subsequent genotyping (Fig. 1). In our opinion, this approach can be effective and should allow obtaining a full STR profile from very low DNA concentrations. Another possible solution, not tested in the present study, could be to replicate the amplification of samples containing very low DNA concentrations. It was suggested that seven or two replicate tests can lead to a more reliable consensus DNA profile [1, 27]. Single amplification of a low amount of DNA can give unreliable results, so replicate testing of low DNA samples should be treated as a general rule not only for low copy samples but also for samples subjected to WGA preamplification.

According to our laboratory validation, for an SGMPlus reaction, in which the heterozygote ratio is above 60 %, stutter values do not exceed 14 %, and no additional peaks are observed; the optimal DNA input is in the range of 0.5–1.25 ng. Input of less DNA usually leads to imbalanced heterozygote ratios and allelic and locus dropouts. The validation for SGMPlus was similar to the one which we described for ProfilerPlus [28]. Thanks to DNA pooling procedure, we were able to obtain a reliable STR profile from a lower amount of DNA.

In the case of degraded DNA, alleles’ recovery, as expected, was remarkably reduced. Almost complete or partial profiles were obtained with RCA-RCA, BL-WGA, PEP, and GenomePlex-preamplified samples. Only GenomePlex and PEP methods produced amplification signals for all DNA concentrations tested. DOP, MDA, and GenomiPhi did not produce amplification signals, despite of the use of very high (100 ng) amounts of degraded template in preamplification (Table 3).

MDA method does not allow the amplification of fragments shorter than 1,000 bp, so our results in the context of MDA and GenomePhi are consistent with previous observations [14, 15]. On the other hand, recent studies on enzymatically degraded DNA using GenomePhi (and GenomePlex) suggested a significant improvement of the quality of degraded DNA profiles after amplification with those commercial kits [18]. However, in our case, the degradation was a random and more natural process leading to significant shortening of DNA fragments, while in the study by Ballantyne et al. [18], loci-specific restriction enzymes were used. On the contrary, other studies on GenomePhi did not produce satisfactory results for DNA isolated from hair and bones [20], as well as from samples stored for several years and subjected to natural degradation process [29].

In the case of PEP method, almost complete profiles were obtained only by using 100 ng of template DNA (90.4 % of expected alleles with the lowest heterozygote’s ratio of 29 %). The effect of weakening amplification signal was visible for longer amplicons. Lower DNA inputs allowed amplification of 14.2 and 4.7 % of expected alleles. As in the case of nondegraded DNA, locus VWA was preferentially amplified, and the ratio of the most and least amplified loci was about 24:1 (D2S1338/VWA) (Table 3).

RCA-RCA produced amplification signals for 10 and 100 ng of template DNA. For 10 ng of input DNA, only single alleles of D3S1358, FGA, and D8S1179 loci were amplified, and the ratio between the smallest and the largest peak of amplification signal was approximately 25:1. For 100 ng of DNA, it was possible to obtain 76.2 % of the expected alleles. RCA-RCA method was originally developed for the analysis of FFPE samples, as an alternative to or improvement of MDA methods (RepliG and GenomiPhi) which are not suitable for analysis of severely degraded DNA [14]. Those results are consistent with our observations (Table 3).

BL-WGA method was originally developed for the analysis of plasma-circulating DNA (apoptotic of <200 bp and necrotic of >5,000 bp DNA) and, similarly to RCA-RCA, as an alternative to MDA (GenomiPhi) which does not replicate fragments shorter than 1,000 bp [15]. No amplification was obtained by using this method for 1 ng of template DNA. For 10 and 100 ng of DNA, we observed incomplete profiles with loss of alleles (e.g., *AMG X*), entire loci (e.g., *FGA*), and presence of extra fractions of DNA (Table 3). The observed heterozygous imbalance reached as high as 9 %, and preferential amplification was observed for *D16S539* and *TH01* loci.

**Fig. 2:**
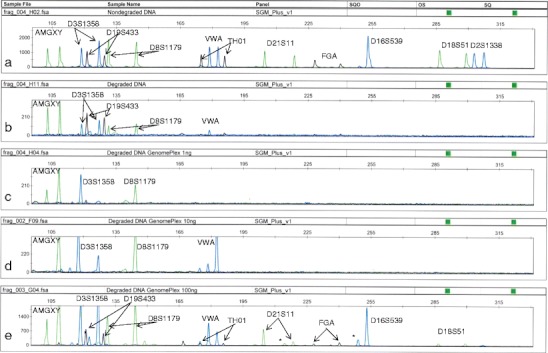
Comparison of SGMPlus profiles of DNA obtained from male muscle tissue. **a** Nondegraded, native DNA; **b** DNA degraded to ca 200 bp; **c**, **d**, **e** degraded DNA preamplified by using GenomePlex amplification of 1, 10, and 100 ng, respectively. *Asterisk* indicates stutters (n-4 fraction)

GenomePlex method produced the best results for degraded DNA, but the number of amplified loci clearly depended on the initial amount of DNA. For 1 and 10 ng of DNA, loss of alleles and loci was observed (amplification of 14.2 and 23.8 % of expected alleles, respectively). For 100 ng of DNA, almost complete profile (90.4 %) was observed; however, shorter DNA fragments, usually amplified preferentially (*AMG Y* and *D16S539*), and stutters up to 22 % were observed. In all cases (1, 10, and 100 ng of input DNA), the allelic imbalance reached ca 30 % (Fig. 2, Table 3).

STR profile recovery is the main and the most important issue in forensic genetics. We focused mainly on degraded DNA analysis, because, very often, the quality of profiles obtained from this kind of DNA is very poor. We demonstrate an analysis of WGA methods in the context of this issue. The comparison of the results for nondegraded and degraded DNA shows that a different approach is needed in their analysis. Application of an appropriate WGA method or tactic (e.g., DNA pooling) may be of great importance in criminal investigation, if it leads to recovery of a key evidence. Similar results were obtained for YFiler loci profiling (data not shown).

### The GenomePlex verification

In general, the GenomePlex method produced the most promising results among the tested WGA techniques, when considering analysis of degraded DNA (200 bp). Therefore, we decided to verify this method using severely degraded DNA (100 bp) and methods of DNA profiling, dedicated for degraded DNA.

### Analysis of Y-chromosome SNPs and mtDNA HVI and HVII regions using DNA degraded to 100 bp

Degraded DNA of muscle tissue (100 bp, 47 days of incubation) and DNA isolated from 4-year-old FFPE muscle tissue were subjected for further analysis. DNA isolated from FFPE tissue showed a very strong degree of degradation, greater than that of the muscle tissue. It also contained small amounts of DNA.

Y-chromosome SNPs with amplicons range of 68–513 bp were subjected to typing with DNA before and after GenomePlex preamplification (Table 4). The shortest amplicon (M173, 68 bp) was successfully amplified before and after GenomePlex preamplification, while for the longest amplicon (M35, 513 bp), the amplification did not occur, neither before nor after being subjected to WGA. The examples of additional loci M9 (164 bp) and YAP (150 bp) demonstrate that the use of WGA methods can lead to restoration of the individual loci destroyed during the degradation process (supplementary material, Fig. S3).

**Table 4:** Amplification results of four Y-chromosome SNP loci for a degraded DNA (100 bp) sample and a FFPE sample before and after GenomePlex preamplification

Y-chromosome SNP	Amplicon length (bp)	DNA 100 bp	GenomePlex DNA 100 bp	FFPE sample	GenomePlex FFPE sample
M173	68	+	+	+	+
YAP	150	+	+	−	+
M9	164	−	+	−	+
M35	513	−	−	−	−

(+) positive amplification, (−) lack of amplification

**Fig. 3:**
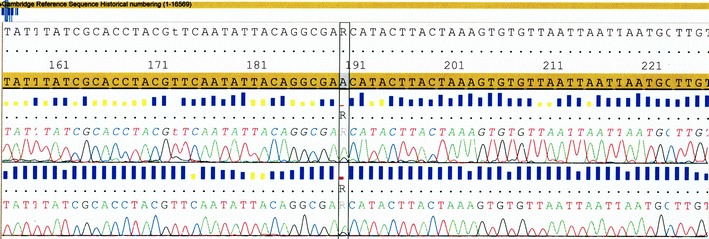
Comparison of HVII regions of nondegraded mtDNA (**a**) and degraded mtDNA preamplified with GenomePlex (**b**). The *box* marks the presence of the A/G heteroplasmy

The aim of the final experiments was to investigate if GenomePlex method can reactivate severely degraded DNA for mtDNA typing. One nanogram of DNA degraded to 100 bp gave negative results when subjected to HVI and HVII sequencing (supplementary material, Fig. S4). After GenomePlex preamplification, complete sequences of both HVI and HVII mtDNA were obtained, identical with the initial sequence of nondegraded DNA, even including some minor DNA features like heteroplasmy (Fig. 3). DNA degraded to 100 bp was reactivated, allowing amplification and sequencing of amplicons longer than 400 bp.

Y-chromosome SNPs analysis is used to examine severely degraded DNA when, for example, identification of heavily degraded remains is required, and paternal relative’s material is provided as a reference sample or, in some criminal cases, with a male suspect. mtDNA analysis is also often required in the case of severely degraded DNA. An example is identification of an exhumed bone subjected to kinship testing in maternal linage. We demonstrate here that application of WGA remarkably improves the possibilities of forensic genetics in this field.

## Conclusions

Seven WGA methods were compared in terms of their possible application for degraded and nondegraded DNA analysis in forensic genetics. The best results for nondegraded DNA were obtained with GenomiPhi and PEP methods. MDA-based GenomiPhi technique is one of the most often used methods in forensic genetics; however, it requires good quality template which reduces its usefulness in this field.

The best results for degraded DNA (200 bp) were obtained with GenomePlex which successfully amplified even severely degraded DNA (100 bp), enabling correct typing of not only Y-SNP loci (100–150 bp) but also mtDNA (~400 bp). Although none of the analyzed methods gave fully satisfactory results, some of them may be very useful in analysis of LCN or degraded DNA in forensic genetics, especially after application of some improvements (sample pooling and replicate DNA typing).

## Electronic supplementary material

Below is the link to the electronic supplementary material.

Figure S1 The control of the in vitro DNA degradation process with 0,5 % agarose gel electrophoresis (SYBR Green I staining). Legend: S—DNA size standard (pGEM® DNA marker; Promega); ND—non-degraded DNA, lines numbered 5, 21, 30, 38 and 47 represent days of DNA degradation (DOC 374 kb)

Figure S2 Quantification of 62 bp long amplicon of *human telomerase reverse transriptase* (*hTERT*) *gene* after 15, 21, 38 and 47 days of biological degradation with Real-Time PCR and Quantifiler Human kit (Applied Biosystems). ND—non-degraded DNA, Numbers describing amplification curves (15, 21, 38, 47) correspond to number of days of tissue (DNA) degradation. (DOC 698 kb)

Figure S3 Result of amplification of YAP and M9 SNP loci for degraded DNA (100 bp) and FFPE sample before and after GenomePlex preamplification. From the left: 1—DNA size marker (pGEM® DNA marker; Promega);, 2—nondegraded DNA, 3—degraded DNA (100 bp), 4—degraded DNA after WGA, 5—nondegraded DNA, 6—FFPE DNA before WGA, 7—FFPE DNA after WGA, 8—DNA size marker, 9—nondegraded DNA, 10—degraded DNA (100 bp), 11—degraded DNA after WGA, 12—nondegraded DNA, 13—FFPE DNA before WGA, 14—FFPE DNA after WGA, 15—DNA size marker. (DOC 465 kb)

Figure S4 Results of HVII mtDNA sequencing obtained for degraded DNA (100 bp) (DOC 232 kb)
