# Supplementary material for: Whole genome amplification of degraded and nondegraded DNA for forensic purposes
Source: Int J Legal Med. 2012 Sep 1;127(2):309–19. doi: 10.1007/s00414-012-0764-9 (PMC3578730; doi:10.1007/s00414-012-0764-9)

Figure S1. The control of the in vitro DNA degradation process with 0,5% agarose gel electrophoresis (SYBR Green I staining). Legend: S – DNA size standard (pGEM® DNA marker; Promega); ND – non-degraded DNA, lines numbered 5, 21, 30, 38 and 47 represent days of DNA degradation


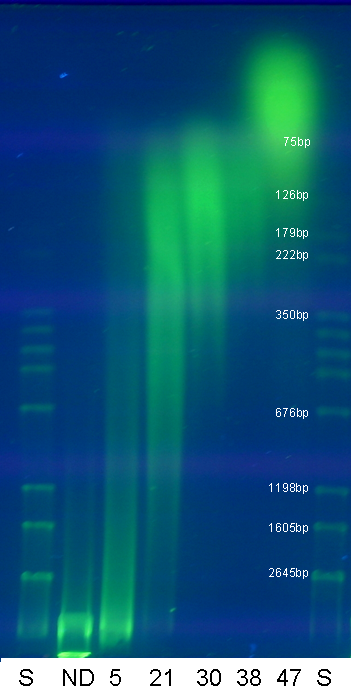

Supplement: Supplementary file 1 — The control of the in vitro DNA degradation process with 0,5 % agarose gel electrophoresis (SYBR Green I staining). Legend: S—DNA size standard (pGEM® DNA marker; Promega); ND—non-degraded DNA, lines numbered 5, 21, 30, 38 and 47 represent days of DNA degradation (DOC 374 kb) [file 414_2012_764_MOESM1_ESM.doc]
