# Supplementary material for: Whole genome amplification of degraded and nondegraded DNA for forensic purposes
Source: Int J Legal Med. 2012 Sep 1;127(2):309–19. doi: 10.1007/s00414-012-0764-9 (PMC3578730; doi:10.1007/s00414-012-0764-9)

Figure S2. Quantification of 62bp long amplicon of *human telomerase reverse transriptase* (*hTERT*) *gene* after 15, 21, 38 and 47 days of biological degradation with Real-Time PCR and Quantifiler Human kit (Applied Biosystems)

ND – non-degraded DNA, Numbers describing amplification curves (15, 21, 38, 47) correspond to number of days of tissue (DNA) degradation.


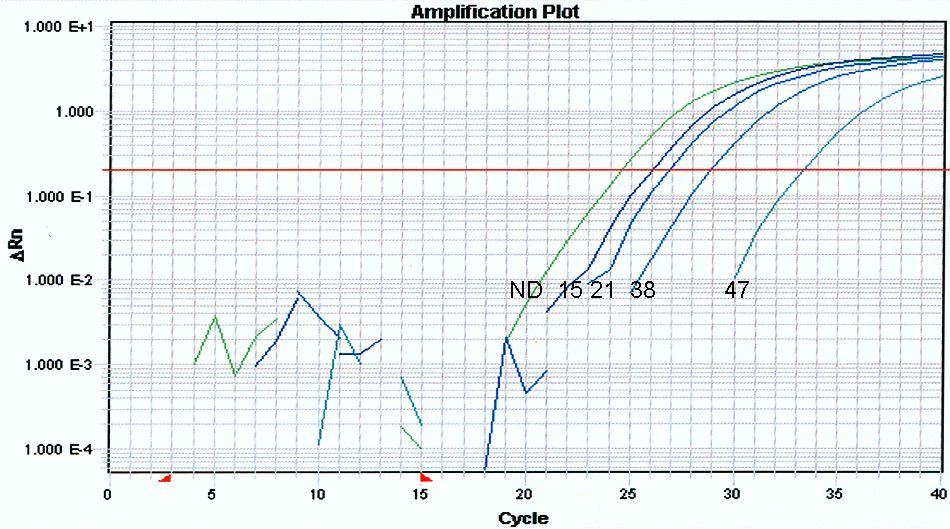

Supplement: Supplementary file 2 — Quantification of 62 bp long amplicon of human telomerase reverse transriptase (hTERT) gene after 15, 21, 38 and 47 days of biological degradation with Real-Time PCR and Quantifiler Human kit (Applied Biosystems). ND—non-degraded DNA, Numbers describing amplification curves (15, 21, 38, 47) correspond to number of days of tissue (DNA) degradation. (DOC 698 kb) [file 414_2012_764_MOESM2_ESM.doc]
