# Supplementary material for: Whole genome amplification of degraded and nondegraded DNA for forensic purposes
Source: Int J Legal Med. 2012 Sep 1;127(2):309–19. doi: 10.1007/s00414-012-0764-9 (PMC3578730; doi:10.1007/s00414-012-0764-9)

Figure S3. Result of amplification of YAP and M9 SNP loci for degraded DNA (100bp) and FFPE sample before and after GenomePlex preamplification. From the left: 1 – DNA size marker (pGEM® DNA marker; Promega);, 2 - nondegraded DNA, 3 - degraded DNA (100bp), 4 – degraded DNA after WGA, 5 – nondegraded DNA, 6 - FFPE DNA before WGA, 7 - FFPE DNA after WGA, 8 – DNA size marker, 9 - nondegraded DNA, 10 - degraded DNA (100bp), 11 – degraded DNA after WGA, 12 – nondegraded DNA, 13 - FFPE DNA before WGA, 14 - FFPE DNA after WGA, 15 – DNA size marker.


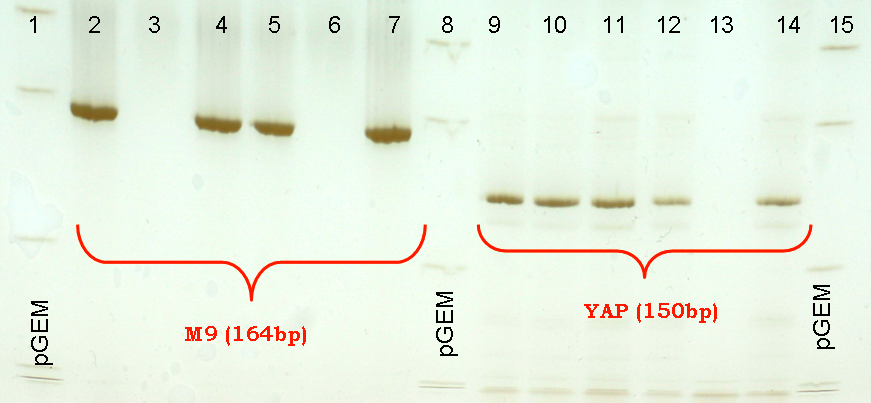

Supplement: Supplementary file 3 — Result of amplification of YAP and M9 SNP loci for degraded DNA (100 bp) and FFPE sample before and after GenomePlex preamplification. From the left: 1—DNA size marker (pGEM® DNA marker; Promega);, 2—nondegraded DNA, 3—degraded DNA (100 bp), 4—degraded DNA after WGA, 5—nondegraded DNA, 6—FFPE DNA before WGA, 7—FFPE DNA after WGA, 8—DNA size marker, 9—nondegraded DNA, 10—degraded DNA (100 bp), 11—degraded DNA after WGA, 12—nondegraded DNA, 13—FFPE DNA before WGA, 14—FFPE DNA after WGA, 15—DNA size marker. (DOC 465 kb) [file 414_2012_764_MOESM3_ESM.doc]
