# Supplementary figures and images for: Whole genome amplification of degraded and nondegraded DNA for forensic purposes
Source: Int J Legal Med. 2012 Sep 1;127(2):309–19. doi: 10.1007/s00414-012-0764-9 (PMC3578730; doi:10.1007/s00414-012-0764-9)

Figure S4. Results of HVII mtDNA sequencing obtained for degraded DNA (100bp)


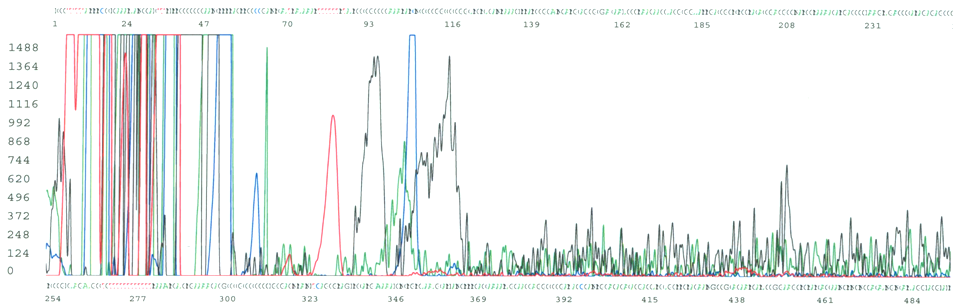

Supplement: Supplementary file 4 — Results of HVII mtDNA sequencing obtained for degraded DNA (100 bp) (DOC 232 kb) [file 414_2012_764_MOESM4_ESM.doc]
